# A mixed-methods evaluation on the efficacy and perceptions of needleless connector disinfectants

**DOI:** 10.1017/ice.2022.72

**Published:** 2023-02

**Authors:** Scott C. Roberts, Curtis A. Hendrix, Lauren M. Edwards, Richard S. Feinn, Richard A. Martinello, Thomas S. Murray

**Affiliations:** 1 Department of Internal Medicine, Yale School of Medicine, New Haven, Connecticut; 2 Department of Infection Prevention, Yale New Haven Health, New Haven, Connecticut; 3 Quality & Safety, Yale New Haven Children’s Hospital, New Haven, Connecticut; 4 Department of Medical Sciences, Frank H. Netter MD School of Medicine, Quinnipiac University North Haven, North Haven, Connecticut; 5 Department of Pediatrics, Yale School of Medicine, New Haven, Connecticut,

## Abstract

**Objective:**

Optimizing needleless connector hub disinfection practice is a key strategy in central-line–associated bloodstream infection (CLABSI) prevention. In this mixed-methods evaluation, 3 products with varying scrub times were tested for experimental disinfection followed by a qualitative nursing assessment of each.

**Methods:**

Needleless connectors were inoculated with varying concentrations of *Staphylococcus epidermidis, Pseudomonas aeruginosa,* and *Staphylococcus aureus* followed by disinfection with a 70% isopropyl alcohol (IPA) wipe (a 15-second scrub time and a 15-second dry time), a 70% IPA cap (a 10-second scrub time and a 5-second dry time), or a 3.15% chlorhexidine gluconate with 70% IPA (CHG/IPA) wipe (a 5-second scrub time and a 5-second dry time). Cultures of needleless connectors were obtained after disinfection to quantify bacterial reduction. This was followed by surveying a convenience sample of nursing staff with intensive care unit assignments at an academic tertiary hospital on use of each product.

**Results:**

All products reduced overall bacterial burden when compared to sterile water controls, however the IPA and CHG/IPA wipes were superior to the IPA caps when product efficacy was compared. Nursing staff noted improved compliance with CHG/IPA wipes compared with the IPA wipes and the IPA caps, with many preferring the lesser scrub and dry times required for disinfection.

**Conclusion:**

Achieving adequate bacterial disinfection of needleless connectors while maximizing healthcare staff compliance with scrub and dry times may be best achieved with a combination CHG/IPA wipe.

Bacterial colonization of needleless connectors is estimated to be the source for up to 50% of central-line–associated bloodstream infections (CLABSIs).^1^ Optimizing disinfection of hubs to reduce bacterial colonization and subsequent biofilm formation remains a key strategy to mitigate the risk of intraluminal bacterial migration, which can result in device-related infections and bacteremia. A key barrier to achieving adequate disinfection is healthcare staff compliance with disinfectant instructions for use. Studies have observed only 10%–31% of clinicians appropriately disinfecting needleless connectors per protocol even when under direct observation.^1,2^

In addition, the optimal methods for disinfection, both in disinfectant type and needleless connector scrub time, remain unclear. Establishing a disinfection method that both reduces microbial colonization and maximizes healthcare staff compliance is ideal. In this mixed-methods study, we compared the efficacy of experimental needleless connector disinfection of 3 products, and we conducted a qualitative nursing assessment of each.

## Methods

### Experimental contamination and disinfection

*Staphylococcus epidermidis* (ATCC 12228)*, Pseudomonas aeruginosa* (laboratory strain PAO1) and *Staphylococcus aureus* (ATCC 25923) were grown overnight in 3 mL of lysogeny broth and were serially diluted. Needleless connectors were inoculated by spreading 5–10 μL of dilute bacteria solution (inocula ranging from 4,150 to 2,000,000) onto the hub, followed by dry times of 30 minutes to 2 hours at room temperature. Inoculum and dry times were purposely varied to evaluate products and scrub times across differing conditions.

We tested 3 products for active disinfection: a 70% isopropyl alcohol (IPA) wipes (the current standard at our institution; ∼6 cm × 2.5 cm; Medium Alcohol Prep Pad, Medline, Northfield, IL,), a 70% IPA cap (Site-Scrub, Bard Access Systems, Salt Lake City, UT), and a 3.15% chlorhexidine gluconate (CHG) with 70% IPA (CHG/IPA) wipe (∼9-cm × 3.5-cm; Prevantics, Professional Disposables International, Orangeburg, NY). All 3 products were applied to the experimentally contaminated Luer lock needleless connector (Baxter ONE-LINK Needle-free IV, Connector, Deerfield, IL), the main needleless connector used in routine clinical care at our institution. Disinfection was performed by both physician and nursing staff members (S.C.R., C.A.H., L.M.E., and T.S.M.) with a consistent and thoroughly applied rotational mechanical friction for the time specified by the manufacturers’ instruction for use (15-second scrub time and 15-second dry time for IPA wipes, 10-second scrub time and 5-second dry time for IPA caps, and a 5-second scrub time and 5-second dry time for CHG/IPA wipes) using a timer. Cultures were obtained immediately after the dry time had elapsed. This process was repeated with Kimwipes (Kimtech Science Kimwipes, Kimberly-Clark Professional, Roswell, GA) wet with sterile water, which were applied for 5, 10, and 15 seconds also using a rotational movement as a control to assess the benefit of mechanical friction alone. As a positive control, experimentally contaminated needleless connectors were cultured after no disinfection or water scrub was performed. Needleless connectors were disinfected by soaking in 70% ethanol for 30 minutes and were dried for at least 15 minutes between inoculations.

To sample the surface of the needleless connector, sterile cotton swabs were dipped in Dey–Engley (D/E) neutralizing broth (Hardy Diagnostics, Santa Maria CA) and wiped on the surface of the needleless connector for 5 seconds in a back-and-forth movement. This swab was then used to inoculate a D/E agar plate, which was incubated overnight at 37°C and bacterial colony-forming unit (CFU) counts were performed the following day.

### Nursing assessment

In total, 35 nurses from 5 intensive care units (ICUs; a cardiothoracic, surgical, medical, step-down, and neurological ICU) were asked to test the 3 products on a needleless connector and to provide feedback in a cross-sectional, observational, qualitative assessment. This study was performed in ICU breakrooms using hands on observations. Semistructured interviews were conducted to assess the percentage of patients the nurses care for who have central lines, needleless connector scrubbing practices, and their perceptions of the scrub practices of their colleagues. The nurses were asked for feedback on each product using open-ended questions and were then asked to rank the 3 products in order of preference for use. Two independent reviewers (T.S.M. and S.C.R.) evaluated the qualitative data for thematic patterns using a grounded-theory approach, and an additional third reviewer (R.A.M.) adjudicated discrepancies.^3^

### Statistical analysis

Data were collected and analyzed using Microsoft Excel version 16.49 software (Microsoft, Redmond, WA). Linear mixed models were used to test for differences in CFU between water and disinfecting methods. Because of nonnormality of the data, CFU were transformed to ranks and the analyses were conducted on the rank scores. Models included fixed effects for disinfecting method, organism, dry time, and inoculum, and a random intercept for experiment. Post hoc paired comparisons with least significant difference were performed following a significant effect for factors with >2 levels (product). Analyses were conducted using SPSS version 27.0 software (IBM, Armonk, NY) and statistical significance was set at an α level of 0.05. This quality improvement initiative was exempt from institutional review board review (Yale University Institutional Review Boards Checklist: 100 CH.9 Clinical Quality Improvement).

## Results

### Disinfection

**Fig. 1.**
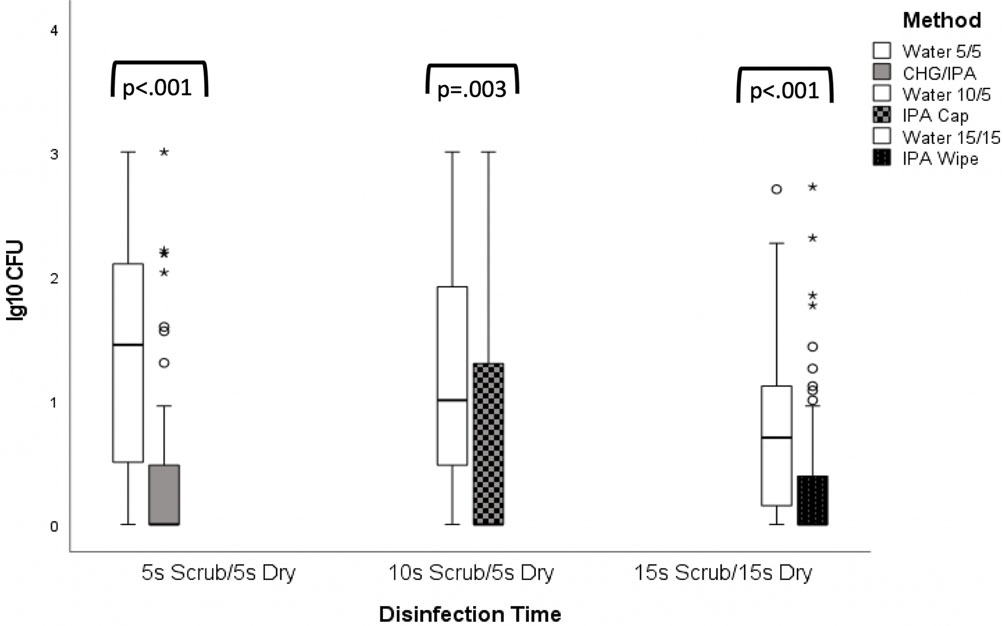
Logarithmic bacterial colony-forming unit (CFU) reduction using a linear mixed model to adjust for covariates (including organism) classified by rank CFU. All 3 products were more effective at reducing bacterial CFU than water controls. When adjusting for initial inoculum, organism, and dry time, no difference in logarithmic bacterial CFU reduction was detected between the IPA wipe and the CHG/IPA wipe. However, higher bacterial CFU counts were noted when the IPA cap was used.

In total, 48 experiments were performed by inoculating varying concentrations of *S. epidermidis* (n = 10)*, P. aeruginosa* (n = 14), and *S. aureus* (n = 24) on needleless connectors. For all 3 organisms, scrub time with sterile water produced a graded response. Increasing times of scrubbing yielded lower microbial CFU for water scrub time of 5 seconds (median, 38 CFU; interquartile range [IQR], 4–189), for water scrub time of 10 seconds (median, 9 CFU; IQR, 2–81), and for water scrub time of 15 seconds (median, 4 CFU; IQR, 0.5–12). When adjusting for the initial inoculum, longer scrub times with sterile water were subsequently associated with less bacterial growth (*P* = .043 for linear trend across the 3 intervals) (Fig. 1).

Low-level growth after disinfection of the needleless connector was observed in needleless connectors disinfected with IPA Site-Scrub caps for *S. epidermidis* (median, 38 CFU; IQR, 0–216) and *S. aureus* (median, 1.5 CFU; IQR, 0–9.25), as well as in needleless connectors disinfected with CHG/IPA wipes for S*. epidermidis* (median, 1 CFU; IQR, 0–19). No other meaningful growth was observed for any other product tested on any other organism combination (Table 1).

**Table 1.** Bacterial Colonization of Needleless Connectors After Experimental Contamination and Scrubbing by Raw CFU Counts

Organism	Inoculum (CFUs)	Dry time^a^	H_2_O (5/5)^b^	H_2_O (10/5)^b^	H_2_O (15/15)^b^	IPA Wipe	IPA Cap	CHG/IPA Wipe
*Staphylococcus epidermidis* (n=10)	60,000 (38,250–297,500)	120 (75–120)	33 (13.75–131)	11.5 (0–138.75)	6 (3–8)	0 (0–1)	38 (0–216)	1 (0–19)
*Pseudomonas aeruginosa* (n=14)	97,500 (43,000–122,500)	120 (120–120)	7 (0–34)	4 (2.5–6.25)	3 (0.5–9.5)	0 (0–0)	0 (0–0)	0 (0–0)
*Staphylococcus aureus* (n=24)	160,000 (90,000–550,000)	120 (120–120)	72.5 (19.5–294)	32 (4–81)	3 (0–43.5)	0 (0–4.5)	1.5 (0–9.25)	0 (0–2)
Overall (n=48)	100,000 (59,250–352,500)	120 (120–120)	38 (4–189)	9 (2–81)	4 (0.5–12)	0 (0–1)	0 (0–8.5)	0 (0–3)

Note. CFU, colony-forming units; H_2_O, sterile water; IPA, 70% isopropyl alcohol; CHG, chlorhexidine. CFU counts are reported as median (interquartile range).

a Dry time in minutes and reflects dry time after organism inoculation.

b Scrub time/dry time in seconds.

After adjusting for the initial inoculum, organism, and dry time, we detected no significant difference between the IPA wipe and the CHG/IPA wipe in bacterial CFU after use (*P* = 0.564). However, higher amounts of bacterial CFU were recovered after use of the IPA cap compared with the IPA wipe (*P* = .008) and the CHG/IPA wipe (*P* = .036) (Fig. 1).

The bacterial reduction of each disinfectant was also compared to mechanical scrubbing for the same duration required for each product according to the manufacturers’ instructions for use but with sterile water instead of disinfectant to evaluate disinfection efficacy when controlling for mechanical scrubbing (Fig. 1). All 3 products significantly reduced bacterial growth when compared to sterile water scrubbing: IPA wipe versus a sterile water 15-second scrub time and 15-second dry time (*P* < .001), IPA cap versus a sterile water 10-second scrub time and 5-second dry time (*P* = .003), and a CHG/IPA wipe versus a sterile water 5-second scrub time and 5-second dry time (*P* < .001). However, for *Staphylococcus epidermidis*, we observed more residual bacteria remaining on the needleless connector after use of the IPA cap compared to a water control, even at shorter scrub and dry times.

### Nursing assessment

We asked 29 nurses how long they scrub the needleless connector before use. Overall, 26 (89.7%) self-reported scrubbing for 15 seconds or longer, and 6 (20.7%) actually reported that they scrub the needleless connector for >30 seconds per each access. Furthermore, 2 (6.9%) stated that they scrub the needleless connector for 10 seconds, and 1 (3.5%) reported that they scrub the needleless connector for 5 seconds. When asked how long they allow alcohol to dry, 21 (72.4%) reported >15 seconds, and 4 (13.8%) reported >30 seconds. Also, 6 nurses (20.7%) reported that they allow the alcohol to dry for 10 seconds, and 2 (6.9%) reported that they allow the alcohol to dry for 5 seconds. When asked what percentage of their nurse colleagues they think perform the full 15-second dry time and 15-second scrub time, the median response was 10% (IQR, 1.5%–50%).

Of the 35 nurses who tested the products, an estimated 80% of patients under their care had central lines (IQR, 71.25%–90%). Also, 24 (68.6%,) preferred CHG/IPA wipes; 9 (25.7%) preferred IPA Site-Scrub caps; but only 2 (5.7%) preferred the status quo of IPA wipes.

A qualitative assessment of the most common themes for favorable or unfavorable opinions for each product identified 3 major themes: (1) the inherent product characteristics, (2) use of the product, and (3) maintenance of the product (Supplementary Table online). Within each theme, various subthemes were apparent. The most common subtheme for why a product was preferred was the shorter time required for scrubbing and drying the needleless connector (n = 16, 45.7%). Many preferred the mechanics of the IPA Site-Scrub caps, in which the needleless connector does not need to be handled directly (n = 12, 34.3%) and the cap covers the entire needleless connector. Many preferred the bigger size of the CHG/IPA wipe (n = 4, 11.4%) and the wetness of the CHG/IPA wipe (n = 4, 11.4%).

Reasons that a product was not viewed favorably include the poor mechanics of the hand motion required for the IPA caps (n = 6, 17.1%). Others commented on the wastefulness of the larger IPA caps (n = 5, 14.3%) and the potential to cause problems at the bedside not seen with wipe products. Some did not like the stickiness of the CHG/IPA wipes (n = 4, 11.4%), one nurse noted that Kelly clamps were required to remove a needleless connector from a connection. The 2 nurses (5.7%) who preferred the IPA wipes reported a sense of familiarity as the main reason, and other nurses criticized the small size of the wipe and the relative dryness when compared to the CHG/IPA wipe. Themes and representative quotes from nursing staff are noted in the Supplementary Table (online).

## Discussion

Disinfection of needleless connectors remains a key strategy in CLABSI prevention. A number of different products have different scrub times within their instructions for use. How these differences impact use in the clinical setting has not been clearly established. All 3 products, IPA wipes, IPA caps, and CHG/IPA wipes, showed significantly greater reductions in bacterial CFUs compared to a sterile water scrub for mechanical bacterial removal. However, both the IPA and CHG/IPA wipes were superior to the IPA cap, showing greater reduction in residual bacteria CFU. Interestingly, in one set of experiments, IPA caps were inferior to sterile water controls for the removal of *Staphylococcus epidermidis* on needleless connectors. This is concerning as *Staphylococcus epidermidis* is one of the most common bacteria to contaminate needleless connectors. Further studies are needed to confirm these findings.

Prior studies performed in clinical settings have found no superior product or scrub time for disinfection of needleless connectors between IPA and CHG/IPA wipes, although notably IPA caps were not tested.^4^ Others have found that a scrub time as short as 5 seconds with CHG to be superior to longer scrub times using IPA products when evaluating needleless connector disinfection in vitro.^5^ Because CHG can have residual bactericidal activity that lasts up to 24 hours, it is possible that the scrub time for CHG products is irrelevant if the entire needleless connector is coated in contrast to other disinfectants, such as IPA, that have no residual activity.^6^ This is important to note because we also found scrub time to be an important determinant in the manual removal of bacterial colonization. When evaluating for the presence of bacteria immediately after scrubbing the needleless connector with only sterile water and no disinfectant, we identified an inverse association between bacterial growth and scrub time.

In our qualitative assessment, it is not surprising that the variable that most commonly determined whether a product was well received by nursing staff was reduced time required for the scrub. Interestingly, although 90% of nurses stated that they perform at least a full 15-second scrub time and 15-second dry time with our current standard of IPA wipes, when asked how long other nurses adhere to this standard, the median prediction was that 10% of nurses achieve this scrub time in a real-world setting. Consequently, initiating use of a CHG/IPA wipe requiring a 5-second scrub time and 5-second dry time poses an attractive option for disinfection of needleless connectors because this reduced scrub time likely improves scrub time compliance without compromising disinfection activity. As staff shortages continue and nurse-to-patient ratios decrease, the potential to save time without compromising the quality of disinfection is appealing. Healthcare systems will need to weigh the benefits of improved compliance and noninferior disinfection against the increased cost of the CHG/IPA wipe compared with the IPA wipe. Nurses had differing views of the IPA cap. Some preferred the mechanics and perceived enhanced disinfection capabilities of using a cap, which fully covers the needleless connector hub without needing to handle the needleless connector directly. Others noted the ergonomics, the environmental waste of a plastic cap compared to a wipe, and the squeaky sound produced during scrubbing to be negatives. As hospital employees and organizations become more environmentally conscious, this new variable may influence purchasing decisions for infection prevention products.^7^ Additional considerations in product use should include the potential toxicities of both CHG and IPA because frequent use can introduce these disinfectants into the bloodstream and may have implications for those with allergies or newborns.

The strengths of this study include a mixed-methods approach in which we queried staff about usability. This led to the identification of themes not typically considered when assessing needleless connector disinfection such as the environmental impact of the product. It also provides insight into staff perception of their own adherence to manufacturers’ instructions for use as well as their colleagues that can inform educational interventions. Also, by utilizing a sterile water control, we were able to quantify the benefits of mechanical scrub on needleless connector decontamination in the absence of a disinfecting product.

This study had several limitations. Laboratory evaluations of product efficacy may have limited generalizability to clinical settings. Additionally, this was a single-center study of nurses working only in ICUs, though staff from 5 separate ICUs were involved. Viewpoints of different product effectiveness may be biased by the current use of IPA wipes and perception of hospital or unit CLABSI rates.

In conclusion, the scrubbing of needleless connectors prior to use is a key component to prevent CLABSIs. In this mixed-methods study, scrubbing with either an IPA wipe, an IPA cap, or a CHG/IPA combination wipe were highly effective for disinfection; however, the IPA and CHG/IPA wipes were superior to the IPA caps. Even though scrubbing the needleless connector with sterile water effectively decreased the bacterial burden on the needleless connector, it was not surprisingly less effective than scrubbing with a germicidal wipe. Nursing staff noted improved confidence with use of the larger, wetter CHG/IPA wipes compared with the IPA wipes, and they expressed concern over the waste associated with the use of IPA caps for scrubbing needleless connectors. Additional research is needed to determine whether the use of IPA impregnated caps with CHG/IPA wipes for scrubbing needleless connectors provides additional microbiological benefit or is associated with reduced risk for bloodstream infections.

## Supplementary material

For supplementary material accompanying this paper visit https://doi.org/10.1017/ice.2022.72. click here to view supplementary material
