# Supplementary material for: A mixed-methods evaluation on the efficacy and perceptions of needleless connector disinfectants
Source: Infect Control Hosp Epidemiol. 2022 Apr 7;44(2):230–3. doi: 10.1017/ice.2022.72 (PMC9929708; doi:10.1017/ice.2022.72)
Supplement: Supplementary file 1 [file S0899823X22000721sup001.docx]

Supplemental Table: Nursing perceptions of Scrub the Hub Products

| Product characteristics | Product use | Product maintenance |
| --- | --- | --- |
| *Familiarity*   - The “familiarity” of using the IPA wipes” is welcome | *Required scrub and dry time**   - Scrub and dry “time is the most important factor” in choosing a preferred product - when using multiple medications in a row “time is most important” - patients could have bad outcomes waiting for a full 15 second scrub and dry during emergencies - “No one does the full 15-second scrub and 15-second dry” | *Storage*   - IPA caps “require more storage” than a pack of wipes - “harder to store” the IPA caps |
| Size   - The IPA wipe is “too small” and feels like “my hands contaminate the hub” during use” - The “bigger [CHG/IPA wipe] covers all” of the NC hubs in contrast to the IPA wipes | *Ergonomics of use**   - IPA caps “feel better” during use - “easiest to use” the IPA caps but they are “squeaky” - “hard to maneuver” the IPA caps when the fingers are holding other items - IPA caps “indented my thumb” | *Wastefulness*   - IPA caps are “bulkier and harder to store”, “most wasteful” of the three products - “plastic waste” of IPA caps pose an environmental hazard |
| *Stickiness of CHG/IPA wipes*   - The NCs are “sticky” after using the CHG/IPA wipes - “stickiness” is bad for the NC hubs | *Perception of disinfection*   - IPA caps “feel like it disinfects better” - IPA caps “disinfect the best because it covers the whole hub” - The only product where “my hands don’t touch the hub and risk contaminating it” is the IPA cap | *Bedside hazard*   - IPA caps “can cause pressure ulcers” and can be “choking hazards” if they are lost and fall on the patient’s bed - I “will find [IPA caps] all over the floor and patient bed” |
| *Dryness of the IPA wipes*   - “IPA wipes feel dryer” which gives the perception of less disinfection |  |  |
